# Actual postoperative protein and calorie intake in patients undergoing major open abdominal cancer surgery: A prospective, observational cohort study

**DOI:** 10.1002/ncp.10678

**Published:** 2021-05-12

**Authors:** Reickly D. N. Constansia, Judith E. K. R. Hentzen, Rianne N. M. Hogenbirk, Willemijn Y. van der Plas, Marjo J. E. Campmans‐Kuijpers, Carlijn I. Buis, Schelto Kruijff, Joost M. Klaase

**Affiliations:** ^1^ Department of Surgery University Medical Center Groningen Groningen the Netherlands; ^2^ Department of Gastroenterology and Hepatology University Medical Center Groningen Groningen the Netherlands

**Keywords:** cancer, energy intake, enteral nutrition, nutrition support, parenteral nutrition, protein intake, surgery

## Abstract

**Background:**

Adequate nutritional protein and energy intake are required for optimal postoperative recovery. There are limited studies reporting the actual postoperative protein and energy intake within the first week after major abdominal cancer surgery. The main objective of this study was to quantify the protein and energy intake after major abdominal cancer surgery.

**Methods:**

We conducted a prospective cohort study. Nutrition intake was assessed with a nutrition diary. The amount of protein and energy consumed through oral, enteral, and parenteral nutrition was recorded and calculated separately. Based on the recommendations of the European Society for Clinical Nutrition and Metabolism (ESPEN), protein and energy intake were considered insufficient when patients received <1.5 g/kg protein and 25 kcal/kg for 2 or more days during the first postoperative week.

**Results:**

Fifty patients were enrolled in this study. Mean daily protein and energy intake was 0.61 ± 0.44 g/kg/day and 9.58 ± 3.33 kcal/kg/day within the first postoperative week, respectively. Protein and energy intake were insufficient in 45 [90%] and 41 [82%] of the 50 patients, respectively. Patients with Clavien‐Dindo grade ≥III complications consumed less daily protein compared with the group of patients without complications and patients with grade I or II complications.

**Conclusion:**

During the first week after major abdominal cancer surgery, the majority of patients do not consume an adequate amount of protein and energy. Incorporating a registered dietitian into postoperative care and adequate nutrition support after major abdominal cancer surgery should be a standard therapeutic goal to improve nutrition intake.

## INTRODUCTION

Surgical procedures cause trauma to body tissue that can lead to the activation of the systemic inflammatory response and an increase in the metabolic demands of the body. In order to maintain postoperative muscle mass and prevent protein catabolism during the increased metabolic demands, adequate protein and energy intake are mandatory.^1^, ^2^ Low skeletal muscle mass and acute muscle loss have been extensively reported as independent risk factors for mortality in critically ill patients admitted to the intensive care unit (ICU).^3^, ^4^, ^5^, ^6^ Maintenance of muscle mass is also necessary to ensure functional recovery after surgery.^1^ In contrast to the growing amount of knowledge about the impact of nutrition and loss of muscle mass on the recovery of ICU patients, there have only been a few studies reporting about these parameters in patients after major surgery. A recent study reported that muscle loss after gastric cancer surgery was associated with higher rates of postoperative complications and longer length of hospital stay.^7^ The European Society for Clinical Nutrition and Metabolism (ESPEN) estimated that the postoperative protein and energy requirements to maintain postoperative muscle mass are 1.5 g/kg/day and 25–30 kcal/kg/day, respectively.^1^

The importance of adequate protein intake in critically ill ICU patients has been shown in a recent study in which a protein intake of >1.2 g/kg during the first 2–4 days of admission to the ICU was associated with lower mortality in critically ill patients with low muscle mass.^3^ In contrast, there are limited studies reporting the actual nutritional protein and energy intake after major abdominal surgery. The main aim of our current study was to prospectively quantify the actual postoperative protein and energy consumption of patients who are undergoing open major abdominal cancer surgery during the first postoperative week.

## METHODS

### Study design

This study is part of the MUSCLE POWER study, an observational, single‐center, prospective cohort study aiming to identify the presence, impact, and risk factors for clinically relevant, surgery‐related muscle loss in patients undergoing major open abdominal cancer surgery at the University Medical Center Groningen in the Netherlands.^8^ Fifty patients who were scheduled for major abdominal cancer surgery based on an underlying malignancy of the liver, pancreas, bile duct, colon, or rectum were included between May 2019 and November 2019.

Exclusion criteria were as follows: emergency surgery, laparoscopic surgery, robotic‐assisted laparoscopic surgery, patients <18 years old, and patients unable to cooperate to give written informed consent.

This study was approved by the Medical Ethics Committee of the University Medical Center Groningen, the Netherlands, and the study protocol was registered within the Netherlands Trial Register ([NTR]; NTR NL7505, version 1.0, February 7, 2019). Written informed consent was obtained from all patients participating in this study.

### Patient characteristics

Demographic data including age, gender, weight, height, medical history, disease characteristics, and prior oncological treatment were prospectively recorded using electronic patient files.

### Outcome measures

#### Nutritional protein and energy intake

Postoperative nutrition management was based on the recommendations of the Enhanced Recovery After Surgery (ERAS) program.^9^, ^10^ Actual daily nutritional protein and energy intake were recorded from the first postoperative day up to 7 days after surgery or up to the day of discharge in case patients left the hospital within the first postoperative week. The amount of protein and energy consumed through oral, enteral, and parenteral nutrition was recorded separately by patients, nurses, and kitchen staff. The amount of protein and calories consumed each day was calculated with a nutrition calculator application (Isala voeding, Isala Development Services, Netherlands Nutrition Center Foundation). Based on the recommendations of ESPEN, protein intake was considered to be insufficient when patients received <1.5 g/kg protein for 2 or more days during the first postoperative week. Energy intake was considered insufficient if patients received <25 kcal/kg for 2 or more days during the first postoperative week.^1^ The same definitions were also used for patients who were discharged within the week after surgery. Nutrition intake in these patients was not recorded after hospital discharge.

### Complications

The occurrence and severity of postoperative complications until 30 days after discharge were registered according to the Clavien‐Dindo classification system.^11^ Grade I complications were defined as any deviation from the normal postoperative course without the need for pharmacological treatment or interventional procedures. Grade II complications were complications requiring pharmacological treatment. Major postoperative complications were classified as grade III (ie, severe adverse events requiring interventional procedures) and grade IV (ie, life‐threatening adverse events requiring intensive care support). Treatment‐related mortality was defined as patient death within 30 days of surgery or during hospital stay (grade V).

### Data analysis

Data were analyzed using SPSS Statistics version 24.0 (IBM Corporation, Armonk, NY, USA). Distribution of the data was assessed with histograms. Continuous variables are presented as mean with SD, and categorial variables as number with proportion (percentage). Hypothesis testing was done using an unpaired *t*‐test or a Mann‐Whitney *U* test for normal or nonnormal distributed data, respectively. The significance threshold was set at *P* < .05.

## RESULTS

### Patient characteristics

Patient and tumor characteristics for the entire cohort are shown in Table 1. Fifty patients were enrolled in this study. Twenty‐eight (28 of 50 [56%]) were female, the mean age was 64 ± 13 years, and the mean body mass index was 27 ± 4 kg/m². Almost half of the patients had a colorectal tumor (21 of 50 patients [42%]), and in nine cases (9 of 50 patients [18%]) metastatic disease was already present during surgery. Twenty‐six patients (26 of 50 patients [52%]) had undergone prior abdominal surgery.

**TABLE 1:** Patient and tumor characteristics

Variable	Data
Age, mean (SD), years	64 (13)
Body mass index, mean (SD), kg/m^2^	27 (4)
Gender, N (%)	
Female	22 (56)
Comorbidity, N (%)	
Hypertension	16 (32)
Cardiac comorbidity	13 (26)
Pulmonary comorbidity	6 (12)
Renal comorbidity	3 (6)
ASA classification, N (%)	
I	2 (4)
II	39 (78)
III	9 (18)
Distant metastases, N (%)	
0	30 (60)
1	7 (14)
X	13 (26)
Location of the tumor, N (%)	
Colorectal	21 (42)
Liver	10 (20)
Pancreas	10 (20)
Bile ducts	8 (16)
Pseudomyxoma peritonei	1 (2)
Prior abdominal surgery, N (%)	
Yes	26 (52)
No	24 (48)
Prior oncologic treatment, N (%)	
Neoadjuvant chemoradiotherapy	11 (22)
Neoadjuvant chemotherapy	1 (2)
None	38 (76)

Abbreviation: ASA, American Society of Anesthesiologists.

Table 2 provides an overview of the different treatment characteristics; major liver resections (14 of 50 patients [28%]) and colon resections (11 of 50 patients [22%]) were most frequently performed. Intestinal anastomoses were made in 19 patients (19 of 50 patients [38%]), and an intestinal stoma was created in nine patients (9 of 50 patients [18%]). Two patients (2 of 50 patients [4%]) had a nontherapeutic laparotomy because of the intraoperative unexpected detection of extensive peritoneal metastases.

**TABLE 2:** Treatment characteristics

	Data, N (%)
Surgical procedure	
Major liver resection	14 (28)
(Sub) total colon resection	11 (22)
PPPD	9 (18)
CRS with HIPEC	4 (8)
Colon and liver resection	3 (6)
(Sub) total pelvic exenteration	2 (4)
Distal pancreatectomy	2 (4)
Partial small‐bowel resection	1 (2)
Partial small‐bowel resection and liver resection	1 (2)
Whipple	1 (2)
Nontherapeutic laparotomy	2 (4)
Intestinal anastomoses	
0	31 (62)
1	18 (36)
2	1 (2)
Stoma postoperatively	9 (18)

*Note*: Major liver resection is defined as a resection of at least three liver segments.

Abbreviations: CRS, cytoreductive surgery; HIPEC, hyperthermic intraperitoneal chemotherapy; PPPD, pylorus preserving pacreatoduodectomy.

During the first postoperative week, the majority of patients (36 of 50 patients [72%]) received oral nutrition only, eight patients (8 of 50 patients [16%]) received enteral nutrition through a duodenal feeding tube, and six patients (6 of 50 patients [12%]) received parenteral nutrition.

Oral nutrition was initiated on the first postoperative day in 17 patients (17 of 50 patients [34%]), and enteral nutrition was initiated on the first postoperative day in seven patients (7 of 50 patients [14%]).

All 50 patients were admitted to the hospital up to the third postoperative day. Five patients (5 of 50 patients [10%]) were discharged on the fourth postoperative day, four patients (4 of 50 patients [8%]) on the fifth day, and seven patients (7 of 50 patients [14%]) were discharged on the seventh day after surgery. The remaining 34 patients (34 of 50 [68%]) were still admitted to the hospital 1 week after surgery.

### Protein and energy intake

Patients were encouraged to start a normal diet with the addition of oral nutrition supplements on the first postoperative day in accordance with the nutrition recommendations of the ERAS program. Patients were provided five daily meals by the hospital food service. Patients consumed their meals in their own rooms and were encouraged to eat sitting at a table.

**FIGURE 1:**
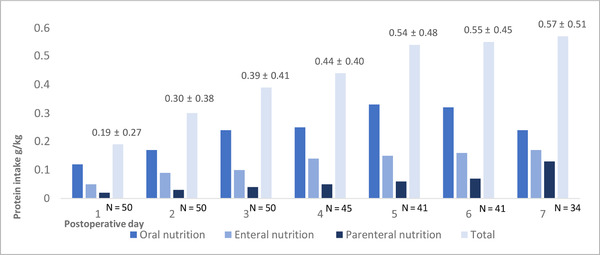
Protein consumption after surgery

The mean daily protein consumption was 0.61 ± 0.44 g/kg/day (ie, 41% consumption of the recommended 1.5 g/kg daily protein intake). Figure 1 shows that during the first 7 postoperative days the daily protein intake gradually increased from 0.19 g/kg/day to 0.57 g/kg/day. Forty‐five patients (45 of 50 [90%]) were not able to consume the recommended amount of protein. Most protein was consumed through oral nutrition (Table 3). Furthermore, more protein was consumed through enteral nutrition via a duodenal feeding tube compared with parenteral nutrition. There was no difference between the mean daily protein consumption of patients who were discharged before the seventh postoperative day and that of patients who were still admitted to the hospital after the first postoperative week (36.36 ± 18.12 g vs 30.08 ± 26.37 g, *P* = .38*)*. No significant differences were found in the daily protein intake between those patients with and without metastatic disease (39.22 ± 34.31 g vs 28.32 ± 20.25 g, *P* = .27).

**TABLE 3:** Daily protein and energy consumption through oral, enteral, and parenteral nutrition

	Daily protein consumption, mean ± SD, g/kg				Daily energy consumption, mean ± SD, kcal/kg			
Postoperative day	Oral nutrition	Enteral nutrition	Parenteral nutrition	Total	Oral nutrition	Enteral nutrition	Parenteral nutrition	Total
1 (n = 50)	0.12 ± 0.20	0.53 ± 0.18	0.02 ± 0.13	0.19 ± 0.27	2.75 ± 4.65	1.15 ± 3.85	0.34 ± 2.40	4.25 ± 5.75
2 (n = 50)	0.17 ± 0.26	0.09 ± 0.28	0.03 ± 0.19	0.30 ± 0.38	4.10 ± 6.15	1.92 ± 5.63	0.62 ± 3.67	6.64 ± 8.09
3 (n = 50)	0.24 ± 0.31	0.10 ± 0.31	0.04 ± 0.21	0.39 ± 0.41	5.45 ± 5.57	2.32 ± 6.49	0.74 ± 3.92	8.51 ± 8.55
4 (n = 45)	0.25 ± 0.27	0.14 ± 0.38	0.05 ± 0.22	0.44 ± 0.40	5.83 ± 6.57	2.92 ± 7.76	0.71 ± 3.32	9.46 ± 7.77
5 (n = 41)	0.33 ± 0.42	0.15 ± 0.39	0.06 ± 0.25	0.54 ± 0.48	8.82 ± 10.11	3.10 ± 8.14	0.94 ± 3. 59	12.87 ± 11.83
6 (n = 41)	0.32 ± 0.39	0.16 ± 0.41	0.07 ± 0.25	0.55 ± 0.45	7.42 ± 9.41	3.30 ± 8.35	1.10 ± 4.00	11.82 ± 10.03
7 (n = 34)	0.24 ± 0.34	0.19 ± 0.46	0.14 ± 0.37	0.57 ± 0.50	6.11 ± 7.61	3.84 ± 9.46	1.73 ± 4.18	12.03 ± 9.74

**FIGURE 2:**
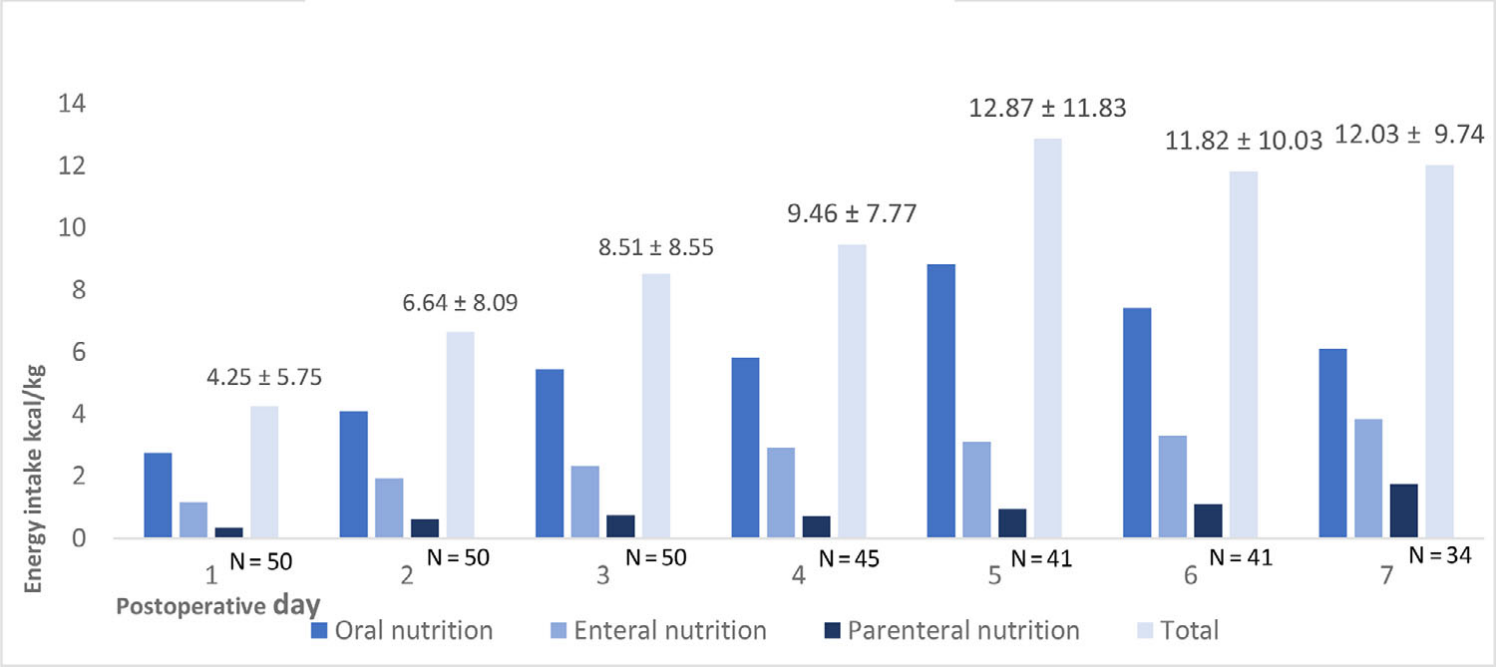
Energy intake after surgery

Energy intake averaged 9.58 ± 3.33 kcal/kg/day, which is associated with only 38% of the recommended 25 kcal/kg daily postoperative energy intake (Figure 2). Forty‐one patients (40 of 50 patients [82%]) did not consume sufficient energy. Similar to the protein consumption, most energy was consumed through oral nutrition (Table 3). The mean energy intake of patients who were discharged early did not differ from that of patients who were still admitted to the hospital after the first postoperative week (803 ± 431 kcal vs 659 ± 520 kcal, *P =* .33). No significant differences were found in the daily energy intake between patients with and patients without metastatic disease (882.11 ± 641.98 kcal vs 624.29 ± 418.82 kcal, *P* = .20).

### Postoperative complications

Table 4 presents the overall postoperative morbidity rates categorized by type and severity of the postoperative complication. No treatment‐related mortality occurred. Ten patients (10 of 50 patients [20%]) had grade I or II complications, and 14 patients (14 of 50 patients [28%]) had grade ≥III complications.

**TABLE 4:** Postoperative complications

Complications	N (%)
Grades I–II	10 (20)
Grade ≥III	14 (28)
Reoperation	5 (10)
Hospital mortality	0 (0)
Grade ≥3	
Gastroparesis	9 (18)
Anastomotic leakage	8 (16)
Electrolyte disorder	6 (12)
Postoperative bleeding	5 (10)
Intra‐abdominal abscess	4 (8)
Wound infection	4 (8)
Pneumonia	3 (6)
Pulmonary embolism	1 (2)
Anemia	1 (2)

*Note*: Electrolyte disorder: serum sodium concentration <135 mmol/L or a serum potassium level <3.5 mmol/L.

All patients with a postoperative complication had insufficient nutritional protein and energy intake days prior to the complication, and postoperative nutrition intake decreased in nine patients (9 of 50 patients [18%]) after the occurrence of a postoperative complication.

Patients with grade ≥III complications consumed less daily protein compared with the group of patients with no complications and patients with grade I or II complications (20.06 ± 21.92 g vs 36.77 ± 22.41 g*, P =* .02).

No statistically significant difference was found between the mean daily energy intake in patients with complications grade ≥III and that of the group of patients without complications or with grade I or II complications (470 ± 525 kcal vs 794 ± 456 kcal*, P* = .06).

## DISCUSSION

This prospective observational cohort study showed that, despite efforts to encourage postoperative nutrition intake, patients did not consume the recommended amount of protein and energy during the first postoperative week after major open abdominal cancer surgery. The mean daily protein intake was 33 g (ie, 0.61 g/kg/day), and the mean energy intake was 732 kcal (ie, 9.6 kcal/kg/day). This resulted in an insufficient protein and energy consumption in 45 [90%] and 41 [82%] of the 50 surgical patients, respectively.

Interestingly, postoperative nutrition management based on the ERAS recommendations was mainly focused on early initiation of oral nutrition and not on the required amount of protein and energy necessary for adequate postoperative recovery.

To the best of our knowledge, only a few studies reported about nutrition and energy intake in patients after major surgery. A prospective observational study, including 40 patients who underwent elective cancer surgery, reported an average protein intake of 0.58 g/kg/day without oral nutrition supplements and 0.72 g/kg/day with oral nutrition supplements within the first 3 postoperative days. These results were similar to our own findings.^12^ Regarding the energy intake, they reported a mean intake of 1025 kcal without oral nutrition supplements and 1310 kcal with oral nutrition supplements during the first 3 postoperative days. In contrast to our study, in which energy consumption averaged 38% of the recommended amount, the authors reported that energy intake reached 60% of the minimum required amount after implementing the ERAS protocol. In another study, including 22 elderly patients who underwent cardiac surgery, higher mean protein intake and energy intake were found during the first 3 postoperative days (0.7 ± 0.3 g/kg/day and 2395 ± 645 kcal/day, respectively).^13^ In comparison, our study population showed lower protein and energy intake; this might be explained by the fact that one could expect an earlier return of the gastrointestinal function after cardiac surgery compared with open abdominal cancer surgery. Despite receiving enteral nutrition, which was initiated on the second postoperative day, 31 patients who underwent pancreatic cancer surgery only consumed 27 g of protein and 588 kcal of energy during the first 2 postoperative weeks.^14^

There are studies that report that adequate protein and energy intake after surgery improve surgical outcomes, including lower risks of late infections and a shorter length of hospital stay.^15^, ^16^ This study found no difference in the protein intake of patients discharged prior to the seventh postoperative day and patients hospitalized during the entire postoperative week.

Patients in this study may have a low nutritional protein and energy intake because they received open abdominal surgery, which enhances surgical trauma compared with laparoscopic procedures. It has been shown that patients undergoing open abdominal surgery have a higher risk for developing severe postoperative malnutrition compared with patients receiving minimal invasive surgery for gastrointestinal cancer.^17^

To improve nutrition intake after major abdominal surgery, the ESPEN and ERAS programs recommend initiating oral or enteral nutrition within 24 h after surgery.^1^, ^10^, ^18^ A meta‐analysis of 11 randomized controlled trials including 1095 patients found that early enteral nutrition improved nutrition status, reduced the risk of postoperative complications, promoted the functional recovery of the digestive system, and shortened the length of hospital stay after gastrointestinal surgery.^19^ Only 17 (34%) patients received oral nutrition within 24 h after surgery, and enteral nutrition was initiated within 24 h after surgery in seven patients (14%). Hence, in order to increase nutrition intake, enteral nutrition should be started earlier in patients who do not tolerate oral nutrition.

Multiple barriers have been shown to affect patients’ nutrition intake when in the hospital. Table 5 shows nutrition barriers and possible measures that could aid in increasing postoperative nutrition intake. First, the feeling of being physically ill and a negative frame of mind negatively affect nutrition intake by decreasing appetite and energy.^20^, ^21^

**TABLE 5:** Measures to increase postoperative nutrition intake

Causes for insufficient postoperative nutrition intake	Measures to increase postoperative nutrition intake
In‐hospital barriers	
Insufficient knowledge regarding nutrition management of the hospital staff	Educational programs regarding the importance of adequate nutrition Providing information about the amount of protein and energy patients require Describing which foods are high in protein and energy
Mandatory fasting for diagnostic procedures and (acute) operations	Reducing periods of fasting during hospital admission
Dietary restrictions ‐ Electrolyte‐restricted diets ‐ Fat‐restricted diets ‐ Cholesterol‐restricted diets	Avoiding unnecessary dietary restrictions as much as possible.
Miscommunications between the hospital staff and patients	Providing clear explanations about which types of food and how much food patients should eat Encouraging kitchen staff and dietitians to talk about nutrition with patients
Patient‐related factors	
Insufficient knowledge regarding postoperative nutrition	Proving information about the amount of protein and energy patients require and describing which foods are protein‐ and energy‐rich by: ‐ Handing out folders with information about nutrition ‐ Hanging posters with pictograms of the recommended food products on the ward ‐ Providing nutrition applications in which patients can record and keep track of the amounts of protein and energy they consume ‐ Face‐to‐face consultations by dietitians
Patient discomfort Physical factors ‐ Nausea ‐ Malaise ‐ Bloating ‐ Ileus/gastroparesis ‐ Pain Psychological factors ‐ Depression ‐ Sadness ‐ Delirium ‐ Forgetfulness	Preventing patient discomfort and managing the physical and psychological factors that negatively affect food intake and recovery
Lack of motivation to eat	Daily motivating patients to consume their required amounts of protein and energy Stimulating patients to eat home‐cooked meals if patients do not like the meals provided by the hospital Encouraging patients to eat while seated at a table unless they are physically restricted

Dietary restrictions, such as sodium‐restricted diets, low‐fat or cholesterol‐free diets, and missed meals because of diagnostic procedures, also cause insufficient nutritional protein and energy intake.^20^, ^22^, ^23^, ^24^ Patients have described missing home‐cooked meals as a barrier for food intake; hence, encouraging consumption of homemade meals could also improve nutrition consumption.^20^, ^25^

Inadequate knowledge, inadequate communication, and misconceptions regarding the importance of nutrition among patients and care providers also cause insufficient nutrition intake.^24^, ^26^ Patients have considered their medical treatment to be more valuable than dietary interventions and did not recognize eating poorly as a problem.^25^

Enhancing patient nutrition knowledge can be achieved by handing out nutrition folders, hanging posters with pictograms of protein‐rich and energy‐rich foods on the ward, or providing patients with digital nutrition applications. Nutrition applications could also help patients keep track of the amount of protein and energy they consume. Providing patients the opportunity to talk about postoperative nutrition with a registered dietitian will increase the nutrition knowledge of patients, which might hopefully lead to an increase in postoperative nutrition intake.

Educational programs for hospital personnel highlighting the importance of adequate nutrition are also necessary to improve nutrition intake. A qualitative study among members of the hospital staff reported that providing education, increasing awareness, and good communication between hospital personnel are required to improve in‐hospital nutrition care.^26^ However, what we learned from this study is that we should incorporate the expertise of a registered dietitian into patient care when measures to increase postoperative oral nutrition fail. A registered dietitian has the knowledge and expertise to optimize postoperative nutritional protein and energy intake. Furthermore, if a patient is unable to adequately consume nutrition orally, then enteral nutrition support should be considered by the healthcare team.

Physical activity is also important to maintain muscle mass and function after surgery. Recent studies have shown that physical inactivity leads to loss of muscle mass and function.^27^, ^28^

### Limitations

The present study has some limitations. Dietary intake was examined by using a nutrition diary that gives an estimation of the nutrition intake. However, measuring the weight of the food consumed would have provided a more accurate measure of the nutrition intake. Hence, the protein and energy intake may be underrepresented or overrepresented in this study. Moreover, nutrition intake was not assessed by a registered dietitian. By utilizing a registered dietitian to assess nutrition intake, a more accurate estimation of protein and energy intake could have been obtained. Furthermore, nutrition intake was not measured after patients were discharged. This could lead to an underestimation of the actual nutrition intake within the first 7 postoperative days, since the patients who were discharged might consume greater amounts of protein and energy at home compared with the patients admitted to the hospital.

Although we reported one of the largest series regarding the actual nutrition intake in patients after major abdominal cancer surgery, the number of patients is still insufficient to perform various subanalyses in the group of patients with grade >III complications in order to identify a direct effect between insufficient nutrition intake and the occurrence of postoperative complications. Furthermore, certain complications, such as anemia, will probably be caused by the chronic illness itself or blood loss during operation rather than by an insufficient postoperative intake. We suspect a clearer answer to these questions in the near future as participants for our earlier mentioned MUSCLE POWER study are still being recruited. In addition, all patients included in this study underwent major surgery in an academic setting. Thus, our study results might not be generalizable to other medical centers.

## CONCLUSIONS

Despite the implementation of ERAS guidelines, the majority of patients in this study had insufficient postoperative protein and energy intake to ensure optimal recovery during the first week after open major abdominal cancer surgery. Incorporating a registered dietitian and adequate nutrition support into postoperative patient care could be a future therapeutic goal to improve the outcome after surgery.

## CONFLICT OF INTEREST

None declared.

## FUNDING INFORMATION

None declared.

## AUTHOR CONTRIBUTIONS

Reickly D. N. Constansia, Judith E. K. R. Hentzen, Carlijn I. Buis, and Joost M. Klaase equally contributed to the conception and design of the research; Marjo J. E. Campmans‐Kuijpers, Rianne N. M. Hogenbirk, and Schelto Kruijff contributed to the design of the research; Reickly D. N. Constansia, Judith E. K. R. Hentzen, and Willemijn Y. van der Plas contributed to the acquisition and analysis of the data; Reickly D. N. Constansia, Judith E. K. R. Hentzen, Rianne N. M. Hogenbirk, Marjo J. E. Campmans‐Kuijpers, Carlijn I. Buis, Schelto Kruijff, and Joost M. Klaase contributed to the interpretation of the data; and Reickly D. N. Constansia and Judith E. K. R. Hentzen drafted the manuscript. All authors critically revised the manuscript, agree to be fully accountable for ensuring the integrity and accuracy of the work, and read and approved the final manuscript.
